# Editorial: Reviews and advances in the molecular mechanisms of breast cancer

**DOI:** 10.3389/fcell.2024.1380475

**Published:** 2024-03-07

**Authors:** A. Redfern, V. Agarwal, S. Alahari

**Affiliations:** ^1^ School of Medicine, University of Western Australia, Perth, WA, Australia; ^2^ Harry Perkins Institute of Medical Research, Perth, WA, Australia; ^3^ Department of Medical Oncology, Fiona Stanley Hospital, Perth, WA, Australia; ^4^ Stanley S. Scott Cancer Center, School of Medicine, Louisiana State University, New Orleans, LA, United States

**Keywords:** breast cancer, invasion, immunotherapy, resistance, CDK4/6 cell cycle inhibitors

This editorial distils the key messages and lessons learned from this article Research Topic to identify future directions for advancing breast cancer (BC) care. First, we have reviews addressing two of the most important questions currently in BC therapeutics; how to extend the gains from immunotherapy to luminal cancers, and how to best treat metastatic luminal cancers following progression on CDK4/6 inhibitor therapy. We then explore the potential of modulating ER beta signalling in triple negative BC (TNBC) and finally look at the biological underpinnings of migration and invasion control to identify therapeutic intervention points.

First, Attalla et al. examine how to optimize the marginal benefits of immune checkpoint inhibitors (ICI) in luminal BC. The authors explore both how to identify luminal responders to ICIs and discuss how to overcome low responsiveness. With current pathological BC classification, only TNBCs are approved for ICI treatment. However, the authors note that using alternative immune-based transcriptomic classifications dividing BC into immune high, medium, and low categories (Galon and Bruni, 2019) may better encompass all who may benefit, including rarer luminal responders. Moving to improving poor luminal responsiveness, observations that tumour-located cytotoxic T cells correlate with benefit suggest that therapies improving tumour lymphocyte infiltration could be beneficial. The potential summarized interventions include agonism of stimulator of interferon genes (STING), reducing immune-suppressive hypoxic conditions through Hypoxia Inducible Factor (HIF) inhibition, and suppressing cytokine gene silencing. Direct manipulation of local immunity through the introduction of cytokine mRNAs or genetically engineered T cells and NK-cells is also covered. Finally, the finding that anti-estrogens may cause immune activation is discussed, leading to the concept of synergy between anti-estrogens, CDK4/6 inhibitors, and ICIs.

This segways into the review of Zhou et al., who examine resistance to CDK4/6 inhibitors, the current standard-of-care for first line metastatic luminal BC management, a question with expanding relevance as adjuvant usage commences. The authors conclude that resistance through attenuation of cell cycle arrest is uncommon, although acquired retinoblastoma protein mutations and CDK 6 over-expression are occasionally implicated. Considering alternate cell cycle entry points, c-myc changes can drive resistance pre-clinically, although clinical targeting is difficult. Encouraging pre-clinical results blocking cell cycle entry through CDK2 (Herrera-Abreu et al., 2016) have led to the development of CDK2 inhibitors. Looking at parallel pathway activation, acquired resistance is linked to AKT and FGFR signalling. Successful trials inhibiting AKT, mTOR, and PI3kinase have led to the recent FDA approval of capivasertib (Turner et al., 2023), with other agents in confirmatory randomised trials. In contrast, early trials of FGFR inhibitors in unselected patients showed modest activity and high toxicity, with trials refocusing on patients with FGFR1 alteration. Encouraging preclinical data also support utilizing androgen receptor (AR) activation in luminal cancers, prompting trials evaluating the selective AR modulator enobosarm as a monotherapy or with abemaciclib. Moving away from signalling modulation, observed immune modulatory changes with CDK4/6 inhibition (Goel S et al., 2017) intertwine nicely with our first review and intimate synergies with ICIs.

Despite the lack of ER-α and progesterone receptor, several other nuclear receptors have relevance in TNBC, introducing prospective new therapeutic targets (Doan et al., 2017) including AR (Kolyvas et al., 2022), thyroid receptor-β (Gu et al., 2015), and ERβ, the subject of our final review (Yan et al.). The role of ERβ is complex, with the authors elucidating the diverse impacts of different splice variants on TNBC biology. ERβ1 has beneficial roles, suppressing oncogenic pathways such as mTOR, TGF-beta, HIF-1α, and AR, suppressing epithelial mesenchymal transition (EMT), maintaining oxidative phosphorylation, and suppressing cholesterol biosynthesis. In contrast, ERβ2, Erβ4, and ERβ5 are oncogenic, enhancing proliferation, invasion, angiogenesis, and EMT through upregulating HIF-1α activity. Although the opposing roles of these variants could make therapeutic targeting difficult, ERβ1 has significantly greater affinity for estradiol than other isoforms, allowing selective stimulation. Both estradiol and the oral ERβ1 agonist S-equol (Lathrop et al., 2020) can inhibit TNBC proliferation and are in clinical trials. Returning to the central theme of tumour immunity in cancer homeostasis, S-equol neoadjuvant treatment also resulted in immune activation in clinical subjects, suggesting that manipulating Erβ1 activity could enhance immune targeting (Lathrop et al., 2020).

Focusing directly on migration and invasion, in our final study Qiu et al. dissect the function of migratory surface structures called lamellipodia, which are important in invasion, with a focus on lamellipodia CD44 overexpression. CD44 is associated with cell adhesion and is known to interact with F-actin, which is involved in lamellipodia function. The authors demonstrate that CD44 overexpression drives increased lamellipodia formation, with enhanced cell migration and EMT. Mechanistically, PI3K/Akt signalling activation leads to overexpression of tissue type plasminogen activator (tPA), which then interacts with LDL receptor related protein 1 (LRP1) to activate downstream NFκB and drive invasion. This provides an opportunity for the inhibition of lamellipodia function at several points through blockade of the tPA/LRP1/NFkB signalling cascade. Pre-clinically, PI3K/AKT inhibition or knockdown of tPA reduced lamellipodia positive cells and attenuated migration.

It is notable that several conceptual therapeutic intersections arise from these outwardly diverse studies. Our initial two reviews independently conclude that synergistic therapeutic value may come from combining CDK4/6 inhibitor blockade with ICI therapy. This concept has already been piloted through the randomised PACE study, where numerically increased progression free survival was observed with the addition of avelumab to fulvestrant and palbociclib, although confirmation is awaited in a larger study (Mayer et al., 2023). Interestingly, TNBC estrogenic activation of ERβ1 led to immune activation in patients (Lathrop et al., 2020), suggesting a reverse strategy where estrogen could enhance ICI function in TNBC. The differing prognostic significance of TILs in luminal BC compared to TNBC (Denkert et al., 2018) suggests that differing immune populations infiltrate these tumours, which may underlie these divergent therapeutic opportunities.

Additionally, several areas of cellular oncobiology repeatedly appear, substantially aligning with the classical hallmarks of cancer (Hanahan and Weinberg, 2011). Beyond the *evasion of immune destruction* discussed above, *reprogramming of energy metabolism* with a shift from oxidative phosphorylation to glycolysis and redirection of glycolysis products into cholesterol biosynthesis was a recurring theme. ERβ1 reversed this effect, with a consequent increase in oxidative phosphorylation and downregulation of cholesterol biosynthesis. Further, we note that upregulation of cholesterol biosynthesis is a key pathway in CDK4/6i resistance (Lanceta et al., 2021) and can also influence the immune microenvironment (Bai et al., 2024). Finally, *invasion and metastasis*, including the related process of EMT, frequently recurs. EMT is upregulated in CDK4/6 inhibitor resistance (Cordani et al., 2023) and is also implicated in modulation of immune response (Williams et al., 2019). CD44, a molecule associated with the EM hybrid state, is convincingly shown to increase lamellipodia-driven invasion by Qiu et al. Finally, ERβ1 may provide a solution in tumours with a suitable isoform balance, having been shown to downregulate HIF-1α and EMT, with a consequent reduction in invasion.

In summary, these studies point to important synergies between immunotherapy and other signalling pathways, with key common cancer behaviours implicated mechanistically that may further guide individualized therapies in the future.

## Scope of submission

This editorial draws together the common elements, biological alignments, and areas of therapeutic promise featured in this Research Topic. The remit for this Research Topic was to explore interactions between malignant cells and the tumour microenvironment that underlie breast cancer (BC) invasion and subsequent progression, with a focus on targeting these interactions for therapeutic benefit. This brief is applied in two timely reviews, addressing probably the two most debated questions currently in BC therapeutics. Firstly, we explore how to extend the remarkable gains observed in more immunologically responsive tumours to BC, particularly traditionally immunologically cold luminal cancers, and then move on to examine current treatment and future management directions for metastatic luminal cancers following progression on CDK4/6 inhibitor therapy. We then shift focus to triple negative BC (TNBC), looking at the complex biology around estrogen receptor beta (ERβ), the frequently overlooked cousin of ER alpha, particularly the role of isoforms in defining malignant behaviour. Finally, we have a detailed dissection of aspects of migration biology, unpicking the central importance of CD44 and the identification of downstream effectors. We conclude by summarizing the areas of common cellular oncobiology that arise from these initially diverse studies and the potential therapeutic interventions they suggest.
